# Ti–Ag–Pd alloy with good mechanical properties and high potential for biological applications

**DOI:** 10.1038/srep25142

**Published:** 2016-04-28

**Authors:** V. Yu. Zadorozhnyy, X. Shi, M. V. Gorshenkov, D. S. Kozak, T. Wada, D. V. Louzguine-Luzgin, A. Inoue, H. Kato

**Affiliations:** 1 National University of Science and Technology “MISIS”, Leninsky prosp., 4, Moscow, Russia; 2 Institute for Materials Research (IMR), Tohoku University, Katahira 2-1-1, Aoba-Ku, Sendai, Japan; 3 National Engineering Research Center for Tissue Restoration and Reconstruction, South China University of Technology, Guangzhou, China; 4 Institute of Multidisciplinary Research for Advanced Materials (IMRAM), Tohoku University, Katahira, 2-Chome, Aobaku, Sendai 980-8577, Japan; 5 WPI- AIMR, Tohoku University, Katahira 2-1-1, Aoba-Ku, Sendai, Japan; 6 School of Materials Science and Engineering, Tianjin University, Tianjin, China; 7 International Institute of Green Materials, Josai International University, Togane, Japan; 8 Department of Physics, King Abdulaziz University, Jeddah, Saudi Arabia

## Abstract

Ti-based alloys containing Ag were produced by tilt-casting method and their properties were studied. Even in its as-cast state, Ti_94_Ag_3_Pd_3_ showed relatively high tensile properties, good electrochemical behavior, and good biocompatibility. The relatively good mechanical properties of the as-cast α-Ti-type Ti_94_Ag_3_Pd_3_ alloy (tensile strength up to 850 MPa and elongation of ~10%) can be explained by its severely deformed, fine crystalline structure. The high biocompatibility of Ti_94_Ag_3_Pd_3_ can be explained by the Ag–Pd interaction, which inhibits the release of Ag ions from the surface. Ag, in combination with Pd has no toxic effects and demonstrates useful antimicrobial properties. The Ti_94_Ag_3_Pd_3_ alloy shows a good potential to be applied as a biomedical implant alloy.

Ti alloys have high specific strength and good corrosion resistance^1^. Recently, we produced some Ti-based alloys with relatively good mechanical properties^2^, including a Ti-3%(at.)Fe-3%(at.)Cu (Ti_94_Fe_3_Cu_3_) alloy with good tensile strength and ductility^3^,^4^. The tensile mechanical properties of this alloy have been improved by dual-axial forging: dual-axial forging it at 1173 K for 15 times. Being treated the alloy exhibited an ultimate tensile strength of about 1200 MPa and an elongation of about 9%.

This alloy contains only small amounts of relatively inexpensive alloying elements, Fe and Cu. Fe play a role of the strong β-stabilizer element. Cu has similar characteristics “β-stabilizer type”, but as a eutectoid in combined additions with other element such as Fe and Cr^5^,^6^. This alloy contains only small amounts of relatively inexpensive alloying elements, Fe and Cu. Fe and Cu play a role of the strong β-stabilizer element. However, Ti-based alloys with Fe and Cu do not have good enough biocompatibility properties for medical applications^7^,^8^,^9^. Thus, substituting Cu for Ag or substituting both Cu and Fe for more biocompatible elements (e.g., Ag and Pd) should make this alloy better in various medical applications.

In the present work, we modify the composition of Ti_94_Cu_3_Fe_3_ by substituting Cu or both Fe and Cu with the noble metals Pd and Ag, which are also late transition metals. We did this to retain the original Ti concentration of 94 at.% and, hopefully, retain the phase composition and mechanical properties of the original alloy.

Palladium (Pd) has already been investigated as an alloying element in medical alloys, as an addition to biodegradable materials^10^,^11^,^12^. The formation of noble Pd-rich precipitates in Fe-based biodegradable materials is expected to induce microgalvanic corrosion, which greatly enhances the degradation rate^13^,^14^,^15^,^16^. But, it should be noted, that these were only preliminary works, and there were no animal testing, only cell culture results. In other works, Ti–Pd alloys have been studied for use in permanent implants^17^,^18^. Ti–Pd alloys are used in applications that require excellent corrosion resistance in chemical processing under mildly reducing or fluctuating reducing/oxidizing conditions^19^. Adding a small amount of Pd to Ti increases corrosion resistance in various media, particularly acids^20^,^21^,^22^,^23^.

Silver is a strong antibacterial agent that is used in colloidal, metallic, and ionic forms. It has excellent broad-spectrum antibacterial properties at low concentrations by interacting with the enzymes and proteins of bacteria, all without cytotoxicity^24^. Thus, Ag is widely used in biomedical applications to inhibit infection^25^. In addition, Ag can damage the cell membrane of bacteria and prevent them from reproducing^24^, and it inhibits the electron transport chain of the bacteria cell, which eventually destroys the bacteria^26^,^27^.

To produce a Ti alloy with better mechanical properties and biocompatibility, we prepared and studied the following Ti-based alloys with added Ag: Ti-3%(at.)Ag-3%(at.)Fe (Ti_94_Ag_3_Fe_3_), Ti-3%(at.)Ag-3%(at.)Pd (Ti_94_Ag_3_Pd_3_) and Ti-6%(at.)Ag (Ti_94_Ag_6_).

## Results

**Figure 1:**
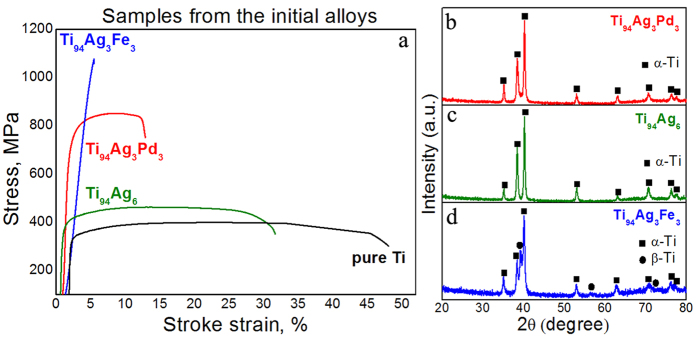
Tensile stress–strain curves of the as-cast alloys with added Ag and as-cast pure Ti (**a**). XRD patterns of as-cast Ti_94_Ag_3_Pd_3_ (**b**) Ti_94_Ag_6_ (**c**) and Ti_94_Ag_3_Fe_3_ (**d**).

The as-cast Ti_94_Ag_3_Pd_3_ alloy had a relatively high tensile strength and ductility (Fig. 1a): across several samples, its average tensile stress was ~850 MPa and its tensile elongation was ~10% (Table 1). These results compare well with the other alloys we studied here (Fig. 1a). The ductility of Ti_94_Ag_6_ was quite high (>30%), but its strength was only ~460 MPa, close to that of pure Ti^31^ (Table 1). For the Ti_94_Ag_3_Fe_3_ alloy, its tensile strength was ~1060 MPa, but its tensile elongation was only 0.5% (Table 1). For comparison, the tensile strength and ductility of as-cast pure Ti are shown in Fig. 1a and Table 1.

Note that Ti_94_Ag_3_Pd_3_ had rather good mechanical properties even in its as-cast state. Thus, this as-cast alloy needs neither special thermo-mechanical nor heat treatment to improve its mechanical properties beyond those of other Ti-based alloys^1^. Its tensile strength (~850 MPa) and elongation (~10%) are relatively high for α-Ti low alloys.

X-ray diffraction (XRD) shows that the as-cast Ti_94_Ag_3_Pd_3_ and Ti_94_Ag_6_ alloys had an α-Ti structure (Fig. 1b,c). Ti_94_Ag_3_Fe_3_ had an α + β-Ti structure (Fig. 1d) because Fe is a good β-Ti stabilizer. The Ti_94_Ag_3_Fe_3_ had relatively high mechanical strength (1060 MPa) likely because it contained the β-Ti phase. Also, Ti_94_Ag_3_Fe_3_ has a small amount of the ω-Ti phase (not detected by the XRD), which explains its relatively low ductility. Table 2 shows the phase compositions of the as-cast alloys, based on XRD.

From the XRD results, Ti_94_Ag_3_Pd_3_ had the major fraction of the α-Ti phase with significantly deformed crystalline lattice. We confirmed this deformation from the lattice parameters, sizes of the coherent-scattering region, and root-mean-squared microstrains of the obtained alloys (Table 2). This relatively large strain in the fine crystalline α-Ti phase structure is the likely cause of the alloy’s good mechanical properties.

**Figure 2:**
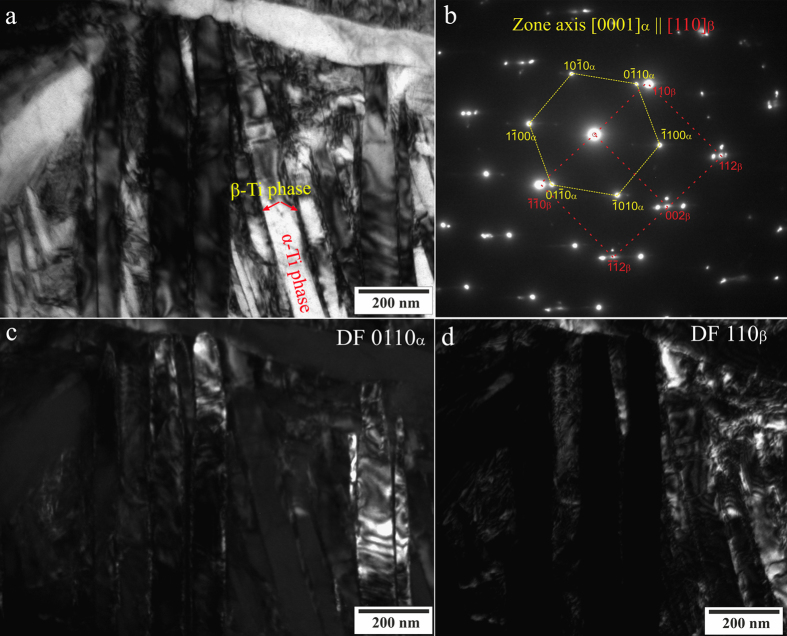
TEM images of as-cast Ti_94_Ag_3_Pd_3_, showing α-Ti and β-Ti grains (a) electron diffraction pattern of the α-Ti and β-Ti grains (b) dark-field TEM image of the α-Ti phase (c) and dark-field TEM image of the β-Ti phase (d).

The strong deformation of the crystalline structure in as-cast Ti_94_Ag_3_Fe_3_ likely came from the formation of a small amount of the β-Ti phase. The XRD pattern of this alloy reveals only the strongest diffraction peak of the β-Ti phase at 2*θ* = ~39° (Fig. 1b), and its volume fraction was impossible to determine because of its low content. However, this phase clearly appears in the TEM image of as-cast Ti_94_Ag_3_Fe_3_ (Fig. 2a). Selected-area electron diffraction patterns from the corresponding areas confirm existence of the α-Ti and β-Ti phases (Fig. 2b). Lamellas of the α-Ti phase also clearly appear in the TEM images, especially in the dark-field image (Fig. 2c), and the β-Ti phase is exposed between the α-Ti lamellas (Fig. 2d).

Han *et al*.^29^ and Takahashi *et al*.^30^ demonstrated a similar distribution of the β-Ti phase between α-Ti phase lamellas. Takahashi *et al*.^30^ showed that Ag is a β stabilizer, securing the β phase by lowering the transformation temperature. However, the α phase has relatively high solubility for Ag and the eutectoid temperature is high, so the β phase may remain upon quenching at any Ag concentration^31^,^32^. For this reason, the β phase cannot be detected by XRD in as-cast Ti–Ag alloys^30^.

**Figure 3:**
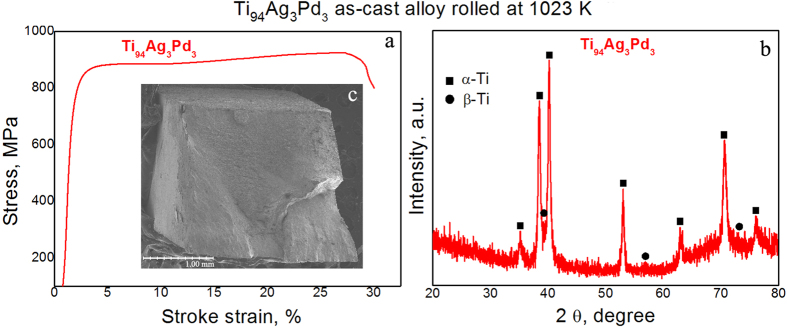
Tensile stress–elongation curves of the Ti_94_Ag_3_Pd_3_ sample rolled at 1023 K in air (a), along with its XRD pattern (b) and fracture surface (c) after a tensile test.

Ti_94_Ag_3_Pd_3_ had comparatively high tensile strength and ductility (Fig. 1a), even in the as-cast state. To reduce the some porosity and break up the large dendritic crystals, in case its occurrence in the as-cast Ti_94_Ag_3_Pd_3_, we performed one-step rolling at 1023 K. After rolling, its tensile strength increased to 920 MPa and its plastic deformation to fracture increased to 27% (Fig. 3a and Table 1), and the β-Ti phase was more visible in its XRD pattern (Fig. 3b). The fracture surface of this rolled alloy shows no any porosity and large dendritic crystals (Fig. 3c).Table 2 shows the phase composition and parameters of the fine crystalline structure of the rolled Ti_94_Ag_3_Pd_3_, revealing that the proportion of the β-Ti phase was close to 15 vol.%.

**Figure 4:**
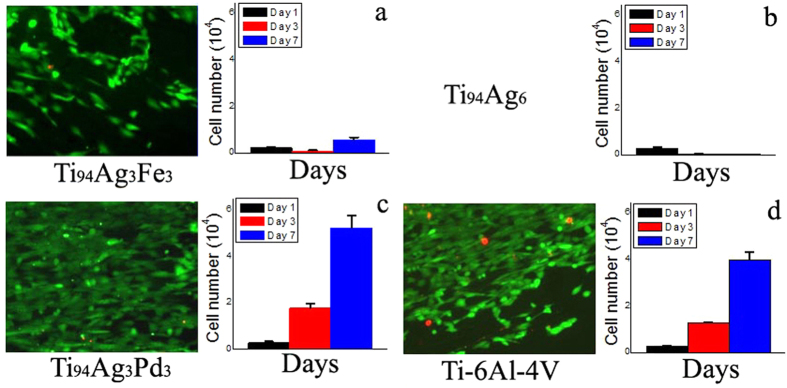
Cell proliferation and phenotypes on the surfaces of the Ag-bearing alloys and industrial Ti-6Al-4V alloy: Ti_94_Ag_3_Fe_3_ (a) Ti_94_Ag_6_ (b) Ti_94_Ag_3_Pd_3_, (c) and Ti-6Al-4V (d). Cell number and live/dead cell staining, magnification: 10×, after 7 days of culture. Significant differences (unpaired Student’s t-test): P < 0.05.

The Ti_94_Ag_3_Fe_3_ and Ti_94_Ag_6_ had poor biocompatibility (Fig. 4a,b). In these alloys, their live cell numbers approached zero because Ag is antiseptic. However, though Ti_94_Ag_3_Pd_3_ contained Ag, it had relatively high biocompatibility (Fig. 4c), just over that of the industrial Ti-6%(wt.)Al-4%(wt.)V (Ti-6Al-4 V) alloy (Fig. 4d), a conventional alloy for biological applications. The live/dead cell numbers obtained during cell proliferation and phenotype tests were 5.221 ± 0.55 for Ti_94_Ag_3_Pd_3_ and 3.986 ± 0.345 for Ti-6Al-4 V.

**Figure 5:**
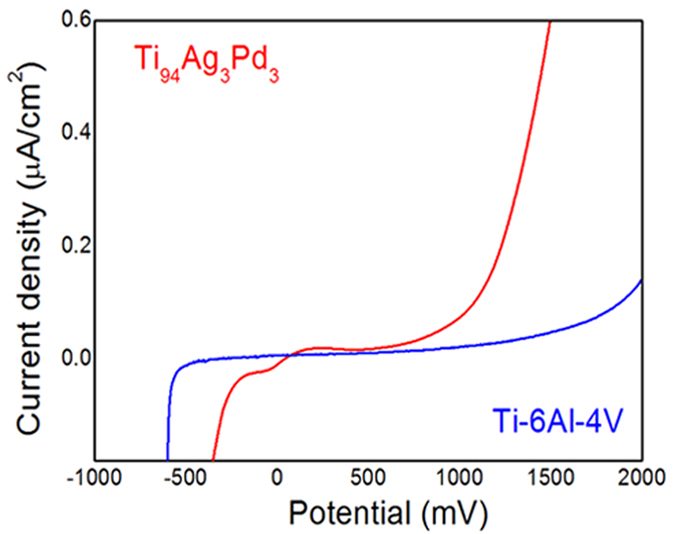
Potentiodynamic anodic polarization plot of Ti_94_Ag_3_Pd_3_ and Ti-6Al-4V, recorded at a scan rate of 2 mV/s.

Permanent implants must be able to withstand potentials of up to +200 mV without visible corrosion (steep slope of the current-voltage characteristic)^33^; a non-negligible current density at 400–500 mV indicates a release of biomaterial ions into surrounding tissue under physiological conditions^34^. Figure 5 shows preliminary electrochemical data for Ti_94_Ag_3_Pd_3_ in a solution of 3% NaCl at 298 K. The passivation layer on the Ti_94_Ag_3_Pd_3_ surface was stable from 0 to +780 mV, displaying current densities of less than ~40 nA/cm^2^, close to values shown in well-known Ti-based alloys^33^,^34^,^35^, including those with added Ag and Pd^29^,^30^,^36^. Reference Ti-6Al-4V alloy exhibited similar electrochemical characteristics (Fig. 5). These results show that the stable passivation layer of Ti_94_Ag_3_Pd_3_ protected the biomaterial in the surrounding tissue under physiological conditions^34^.

**Figure 6:**
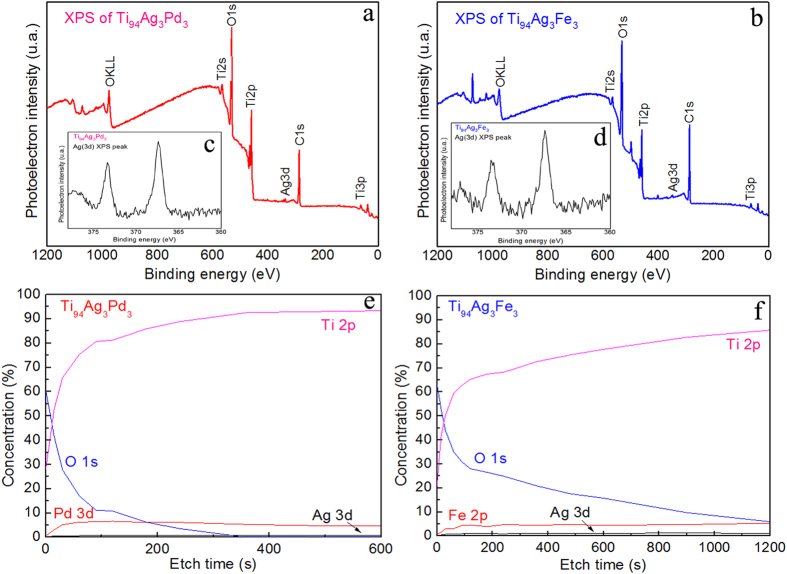
XPS spectra of Ti_94_Ag_3_Pd_3_ (**a**) and Ti_94_Ag_3_Fe_3_ (**b**); narrow-scan spectra of the Ag (3d) XPS peak for Ti_94_Ag_3_Pd_3_ (**c**) and Ti_94_Ag_3_Fe_3_ (**d**); depth-dependent elemental distributions for Ti_94_Ag_3_Pd_3_ (**e**) and Ti_94_Ag_3_Fe_3_ (**f**) as a function of etch time.

To ensure that the Ti_94_Ag_3_Pd_3_ had sufficient biocompatibility and electrochemical behavior, even with a small amount of Ag, we performed X-ray photoelectron spectroscopy (XPS) to compare Ti_94_Ag_3_Pd_3_, which has relatively high biocompatibility, and Ti_94_Ag_3_Fe_3_, which has poor biocompatibility. Using XPS, we quantitatively determined the surface components and compositions of the Ti_94_Ag_3_Pd_3_ and Ti_94_Ag_3_Fe_3_ (Fig. 6). The two alloys exhibited similar XPS spectra (Fig. 6a,b), disregarding the peaks from Pd and Fe. Both alloys contained various Ti oxides, such as TiO_2_ at 458.7 eV and Ti_2_O_3_ at 457.4 eV. The narrow scan spectra of Ag for Ti_94_Ag_3_Pd_3_ and Ti_94_Ag_3_Fe_3_ were also similar (Fig. 6c,d). The splitting of the Ag 3d doublet is 6.0 eV, implying the presence of metallic Ag^37^. However, Ti_94_Ag_3_Pd_3_ had a thinner oxide layer (Fig. 6e) than Ti_94_Ag_3_Fe_3_ one (Fig. 6f). Also, the Ti_94_Ag_3_Fe_3_ surface contained more Ag (more than 1 at.%) than the Ti_94_Ag_3_Pd_3_ one (less than 1 at.%), which is likely an important reason why Ti_94_Ag_3_Fe_3_ had poor biocompatibility.

## Discussion

Overall, our most interesting results are the relatively good mechanical properties of the as-cast Ti_94_Ag_3_Pd_3_ and its unusual biocompatibility regardless of its Ag content.The relatively good mechanical properties of the as-cast Ti_94_Ag_3_Pd_3_ alloy with the α-Ti phase could be partly explained by its fine crystallographic structure (Tab. 2) and the relatively large internal strain of its crystalline lattice (in contrast to the high deformation of lattice parameters caused by the small amount of the β-Ti phase). In this alloy, lamellas of the α-Ti phase appear between the β-Ti layers, and such a layered structure likely generates a relatively large internal strain in the crystalline lattice of the α-Ti phase. We plan to study this effect more in the future. The mechanical properties of Ti_94_Ag_3_Pd_3_ are very similar to or even better than those of the Ti-based alloys with similar chemical compositions shown by Niinomi^38^.

The relatively good biocompatibility of Ti_94_Ag_3_Pd_3_ is quite unusual. Among metals with antimicrobial activity, Ag has garnered interest because it has both good antimicrobial activity and relatively low toxicity^39^. For example, Hardes *et al*.^40^,^41^ showed that silver-coated megaendoprosthesis effectively reduce infections after artificial colonization in an animal trial. In particular, they examined the Ag concentration in the blood and surrounding tissues and documented possible local and systemic side effects in patients treated with a silver-coated megaprosthesis^40^. They also demonstrated that Ag coating was applied to suppress sepsis and infections in the surrounding bone and tissue after prosthesis implantation^40^. However, they noted that if Ag is applied directly to the Ti surface, then the antimicrobial Ag ions cannot dissolve, preventing their sustained release^40^.

Hardes *et al*.^40^ also mentioned that, to drive a release of Ag ions as the anode, a cathode made of a more-noble metal is necessary. Thus, they used a 0.2-mm-thick Au layer between the Ti–V prosthesis and the Ag coating to enable sustained release of Ag ions in the periprosthetic tissue and to prevent progressive corrosion^40^. They^40^ and others^42^,^43^ found that Ag displays bactericidal activity at concentrations as low as 35 ppb without any toxic effects to mammalian cells^42^. Silver-coated materials for external fixation devices were also presented in the work of Bosetti *et al*.^44^. There are many other examples of Ag-containing alloys suitable for bio-applications. For example, Zheng *et al*.^45^ presented a biomedical TiNi shape memory alloy containing 1.4 at. % Ag. This alloy combines antibacterial activity and the shape memory effect. The authors also mentioned slow release kinetics of Ag ions from the Ti-Ni-Ag alloy^45^.

It should be noted, that in some dental alloys, Ag is usually used to obtain relatively good mechanical properties and to substitute (in chemical composition) more expensive elements (like Au or Pt). However, as shown in Fig. 4a,b, Ti_94_Ag_3_Fe_3_ and Ti_94_Ag_6_ alloys containing Ag have a relatively low biocompatibility or even absence of it. Thus, the addition of Ag does not necessarily guarantee good biological compatibility. Usually, for such a kind of alloys containing Ag, some operations are necessary, which induce the depletion from the surface layer in Ag and the preparation of a special oxide layer. For example, it was shown for the dental Ti-Ag alloys in the works of Zhang *et al*.^46^,^47^. The oxide layer in those works consisted mainly of the TiO_2_ film. In the work of Hou *et al*.^48^, passivating films from the TiO_2_ and TiO_3_ oxides were generated on the porous Ti-Ag-based alloys in low vacuum condition. The generated oxides protect the prepared alloy from corrosion and are good for biomedical applications. Therefore, the modifications of the surface layer in case of Ti-based alloys with Ag addition are rather common practice.

As for the XPS results, Yan *et al*.^24^ reported that the binding energies of the Ag (3d_5/2_) core level for Ag, Ag_2_O, and AgO are 368.6, 368.2, and 367.8 eV, respectively. Similar binding energies of Ag (3d_5/2_) have been shown previously^49^,^50^. In our case, for both alloys, the peak maximum appeared at the core level of ~367.6 eV, which is close to the AgO core level (Fig. 6c,d). The peak should experience little widening or shifting to a higher binding energy because there is little Ag_2_O in both alloys. Thus, the Ag oxides found on the surfaces of Ti_94_Ag_3_Pd_3_ and Ti_94_Ag_3_Fe_3_ are similar. This means the Ag^+^ ions should successfully release in both cases, as shown by Pratten *et al*.^42^, even though the Ag concentrations in their surface layers are very different.

Note also that the materials obtained here had oxide layers with different thicknesses: the oxide layer on the Ti_94_Ag_3_Fe_3_ alloy was more than twice as thick as that on the Ti_94_Ag_3_Pd_3_ one (Fig. 6e,f). Even with a thicker oxide layer, Ti_94_Ag_3_Fe_3_ has rather poor biocompatibility, which supports the belief that the relatively high biocompatibility of Ti_94_Ag_3_Pd_3_ can be explained only by the presence of Pd.

Pd is a more noble metal than Ag, so it serves as a cathode material to inhibit the release of Ag ions. A similar effect has been shown previously^40^,^41^ on an Au layer deposited between an implant surface and an Ag coating. Thus, the Ag concentration on the Ti_94_Ag_3_Pd_3_ surface is as high (Fig. 6e) as that on the Ti_94_Ag_3_Fe_3_ surface (Fig. 6f). These reasons explain why Ti_94_Ag_3_Pd_3_ is not very toxic and has rather good biocompatibility (Fig. 4c) compared to Ti_94_Ag_3_Fe_3_ (Fig. 4a) and Ti_94_Ag_6_ (Fig. 4b).

In conclusion, based on previously studied Ti_94_Fe_3_Cu_3_, we produced as-cast Ti_94_Ag_3_Pd_3_ that also has the α + β-Ti structure with a somewhat larger phase fraction of α-Ti. Ti_94_Ag_3_Pd_3_ had a relatively high tensile strength (up to 850 MPa) and acceptable ductility (elongation of ~10%), even in its as-cast state. After ordinary rolling at 1023 K in air, its tensile strength and plastic elongation increased to 920 MPa and 27%, respectively. The Ti_94_Ag_3_Pd_3_ showed acceptable biocompatibility (high level of cell proliferation and phenotypes).

The relatively good biocompatibility of Ti_94_Ag_3_Pd_3_ can be explained by the Ag–Pd interaction. Some kinds of cathode-anode clusters are expected to form, and these clusters control the release kinetics of Ag ions from the surface and favour good biocompatibility and high electrochemical behavior.

Our Ti_94_Ag_3_Pd_3_ alloy shows good potential to be applied as a biomedical implant alloy for the orthopedic surgery and other medical applications because it is simple preparation by casting and relatively good mechanical properties even in the as-cast state, and because the Ag empowers it with antimicrobial properties.

## Methods

### Alloy preparation

Rods of Ti_94_Ag_3_Pd_3_, Ti_94_Ag_6_, and Ti_94_Ag_3_Fe_3_ alloys, 6 mm in diameter and ~50 mm in length, were fabricated by arc-melting mixtures of the pure metals in an Ar atmosphere, purified by Ti getter, and tilt casting them into a Cu mold. Before casting, the ingots were turned over and re-melted five times to ensure compositional homogeneity.

### Analysis of crystalline structures and phase compositions

The atomic structures of the alloys were examined by X-ray diffraction with monochromatic Cu Kα radiation. Lattice parameters and phase compositions were determined with an accuracy of ±0.0001 nm and ±5%, respectively. The dimensions of crystallites in the samples were determined, with an accuracy of ±5 nm, by examining the diffraction peak width.

The microstructures of the ingots were examined with a scanning electron microscope (SEM) with an attached energy dispersive X-ray spectrometer (EDS) at 15 kV.

The samples were also examined with a transmission electron microscope (TEM; JEOL JEM 2010) at 200 kV with an attached energy dispersive X-ray spectrometer (resolution ~0.1 keV). The TEM samples were prepared mechanically (thinned to 10 μm) and then by ion polishing (thinned to electron-beam transparency). To avoid damaging the specimens, the ion-beam energy was kept as low as 2 keV.

The depth profiles of the oxide films were obtained by using X-ray photoelectron spectroscopy (XPS; Axis ultra DLD, Kratos), using a beam size and spot diameter of 300 × 700 μm and 2 mm, respectively. Step sizes of 1 and 0.1 eV were used in the survey and regional scans, respectively. Each regional scan was swept about 10 times. All binding energies given here are relative to the Fermi level, *E*_F_, and all the spectra were obtained using incident monochromatized Al Kα X-rays (energy = 1486.61 eV). Depth-dependent XPS data were acquired by sputtering the surface layers with Ar ion beam. Chemical depth profiles were obtained by alternating sputtering and spectrum acquisition. The XPS profiles at each polishing depth were analyzed using built-in software.

### Rolling and mechanical testing

One-step rolling was performed at 1023 K in air. The rolling reduced the dimensions of the samples by 60–70%.

The tensile mechanical properties were measured at room temperature with a standard mechanical testing machine at a strain rate of 5 × 10^−4 ^s^−1^. The dimension of each tensile sample was 33 mm in length and 2 × 2 mm in rectangular cross-section. The stroke strain was measured using a strain gage (KYOWA DPM-912A) attached to the gage section of the testing specimen.

### Cell culture, seeding, and proliferation

Osteoblasts (hFoB 1.19 ATCC, US) were maintained in DMEM:HAM F12 media with 10% (v/v) fetal bovine serum (FBS) and 1% penicillin/streptomycin. Typically, 100-μl cell suspensions (1 × 106 cells per ml) were seeded on the sample surfaces (cylinders, 1 cm in diameter and ~2 mm in thickness) placed on a cell culture plate. After 1 h, the cells had adhered to the samples, and another 800 μl cell suspension was added to each well and then cultured for 7 days. After 1 day, 3 days, and 7 days of culturing, the cell number was determined using a cell counting kit (Cell Counting Kit-8, Sigma-Aldrich, USA) in accordance with the manufacturer’s instructions. The samples were preliminarily sterilized by immersion in 75% ethanol for 30 min. They were then washed five times with sterilized deionized water.

The live and dead cells were distinguished by using a cell viability kit (Live/Dead Kit, Invitrogen, USA). The kit stained the live cells to be green and the dead cells to be red. The data are presented as a standard error of the mean. The paired means were compared using unpaired Student’s t-tests. P-values of <0.05 were considered to be statistically significant. The numerical P-values are shown in the legend of Fig. 4a–c.

### Electrochemical behavior

A three-electrode cell, built for this research, was used for all the electrochemical tests. The casing of the electrochemical cell was made from a Pyrex glass flask. A Ti_94_Ag_3_Pd_3_ electrode and an Ag/AgCl reference electrode were used. The Pt counter electrode (Pt-CE) was placed on the upper lid, separated by 2 mm from the Ti_94_Ag_3_Pd_3_ electrode and the counter electrodes. The working distance between the electrodes on the upper and the lower lids was 3 mm. The Ag/AgCl reference electrode (diameter of 0.3 mm) and the Pt counter electrodes (diameters of 0.5 mm) were made from Ag and Pt wires, respectively. The working surface area of the Ti_94_Ag_3_Pd_3_ electrode was 0.06 cm^2^.

Potentiostatic polarization tests were conducted using a potentiostat (VersaSTAT4, Princeton Applied Research) at room temperature. Two different saline solutions were prepared for the corrosion test: 3% NaCl and saturated NaCl. This 3% NaCl solution is regularly used to estimate corrosion characteristics. Before testing, the prepared solutions were bubbled with nitrogen gas for 15 min to remove dissolved oxygen. To ensure reproducible results, we performed three experiments under the same conditions, maintaining a scan rate of 2 mV/s. We report the second polarization curve obtained from each set of measurements.

Upon completing a series of polarization measurements, we carefully cleaned and dried the Ti_94_Ag_3_Pd_3_ electrode surface on the lower lid, the cell itself, and the counter electrodes on the upper lid. The electrolyte filling level of the Ag/AgCl reference electrode changed insignificantly during potentiostatic polarization measurements. All electrochemical measurements were conducted at room temperature.

## Additional Information

**How to cite this article**: Zadorozhnyy, V. Y. *et al*. Ti-Ag-Pd alloy with good mechanical properties and high potential for biological applications. *Sci. Rep.* **6**, 25142; doi: 10.1038/srep25142 (2016).

## Figures and Tables

**Table 1:** Mechanical properties of the Ag-added alloys and pure Ti, as-cast and after rolling.

Alloys composition	σ_U_, MPa	σ_0.2_, MPa	δ,%	E, GPa
Ti_94_Ag_3_Pd_3_	850 ± 30	760 ± 40	10 ± 2	90 ± 10
Ti_94_Ag_3_Pd_3_ (rolled at 1023 K)				
Ti_94_Ag_6_	460 ± 30	370 ± 40	30 ± 2	100 ± 10
Ti_94_Ag_3_Fe_3_	1060 ± 20	1050 ± 20	0,5 ± 1	105 ± 10
pure Ti (as-cast state)	400 ± 50	320 ± 40	50 ± 5	110 ± 10
Ti-6Al-4V^28^ (Annealed rod)				

σu – Ultimate tensile strength. σ_0.2_ – Yield strength, δ – Ductility. E - Elastic modulus.

**Table 2:** Alloy composition, phase composition, lattice parameters, coherent-scattering region size, and root-mean-squared microstrains of the Ag-bearing alloys, obtained from XRD measurements, and those parameters for pure Ti, given for comparison.

Alloys composition	Phase composition, vol. %	Lattice parameter, nm	Coherent-scattering region size, nm	Root-mean square microstrain, %
Ti_94_Ag_3_Pd_3_	α-Ti,	a: 0.2896c: 0.4596	25	0.52
Ti_94_Ag_3_Pd_3_ (rolled at 1023 K)	α-Ti, 85β-Ti, 15	a: 0.2916c: 0.4628a: 0.323	20 10	0.025 0.432
Ti_94_Ag_6_	α-Ti, 100	a: 0.2894c: 0.4598	40	0.267
Ti_94_Ag_3_Fe_3_	α-Ti, 70β-Ti, 30	a: 0.2901c: 0.4601a: 0.3178	35 20	0.287 0.268
pure Ti	α-Ti, 100	a: 0.2903c: 0.4602	50	0.16
